# p73-Binding Partners and Their Functional Significance 

**DOI:** 10.1155/2010/283863

**Published:** 2011-01-13

**Authors:** Toshinori Ozaki, Natsumi Kubo, Akira Nakagawara

**Affiliations:** ^1^Laboratory of Anti-tumor Research, Chiba Cancer Center Research Institute, Chiba 260-8717, Japan; ^2^Laboratory of Innovative Cancer Therapeutics, Chiba Cancer Center Research Institute, 666-2 Nitona-Cho, Chuo-ku, Chiba 260-8717, Japan

## Abstract

p73 is one of the tumor-suppressor p53 family of nuclear transcription factor. As expected from the structural similarity between p53 and p73, p73 has a tumor-suppressive function. However, p73 was rarely mutated in human primary tumors. Under normal physiological conditions, p73 is kept at an extremely low level to allow cells normal growth. In response to a certain subset of DNA damages, p73 is induced dramatically and transactivates an overlapping set of p53-target genes implicated in the promotion of cell cycle arrest and/or apoptotic cell death. Cells undergo cell cycle arrest and/or apoptotic cell death depending on the type and strength of DNA damages. p73 is regulated largely through the posttranslational modifications such as phosphorylation and acetylation. These chemical modifications are tightly linked to direct protein-protein interactions. In the present paper, the authors describe the functional significance of the protein-protein interactions in the regulation of proapoptotic p73.

## 1. Introduction

For a long time, *p53* has been believed to be a solitary gene. This classical point of view has been challenged by a discovery of novel human p53 homologues termed p73 and p63 [1–3]. Thus, p53 family is composed of p53, p73 and p63. p73 contains an NH_2_-terminal transactivation domain (TA; amino acid residues 1–54), a central core sequence-specific DNA-binding domain (DB; amino acid residues 131–310), an oligomerization domain (OD; amino acid residues 345–380) and a COOH-terminal sterile *α* motif (SAM; amino acid residues 484–549) domain [1]. Among them, the central core sequence-specific DNA-binding domain is highly conserved (more than 60% amino acid sequence identity) across the family, and p53 lacks the COOH-terminal SAM domain which is involved in the protein-protein interaction [4]. As expected from the structural similarity between p53 and p73, p73 has an ability to transactivate an overlapping set of p53-target genes implicated in cell cycle arrest and/or apoptotic cell death such as *p*21^WAF1^, *MDM2*, *BAX*, *PUMA,* and *NOXA* [5–7]. Indeed, forced expression of p73 induced cell cycle arrest and/or apoptotic cell death in certain cancerous cells [1, 8, 9]. 

 Since *p73* was mapped at human chromosome 1p36.3 where genomic aberrations are frequently observed in a variety of tumors [10–14], it is likely that *p73* could be one of the classical Knudson-type tumor-suppressor genes. To address this issue, the extensive mutation search for *p73* was carried out. In a sharp contrast to *p53* bearing loss of-function mutations in 50% of human tumors [15, 16], *p73* was rarely mutated in human tumors [17], suggesting that *p73* is not a Knudson-type tumor-suppressor gene. In support with these results, initial genetic studies revealed that *p73*-deficient mice do not develop spontaneous tumors [18]. *p53*-deficient mice underwent spontaneous tumor development, mainly sarcomas and lymphomas [19]. Of note, Flores et al. demonstrated that p73 is required for p53-dependent apoptotic cell death, indicating that p73 is one of the essential coactivators for p53, and these observations also emphasized the functional importance of p73 in the regulation of DNA damage-induced p53-dependent proapoptotic pathway [20]. Subsequent genetic studies revealed that mice harboring hemizygous *p73* develop spontaneous tumors, and their spectrum is quite different from that of *p53*-deficient mice [21]. Based on these results, p73 has been considered to be one of the critical tumor-suppressors, although *p73* was infrequently mutated in human primary tumors. 

 Extensive expression studies demonstrated that *p73* encodes at least seven alternative splicing variants with different COOH termini (p73*α*, p73*β*, p73*γ*, p73*δ*, p73*ε*, p73*η*, and p73*ξ*), termed the TA variants [1, 22–24]. Since these splicing variants contain the intact NH_2_-terminal transactivation domain, they have the varied transcriptional potential. Until now, each TA variant-specific biological function remains unclear. In addition to COOH-terminal splicing variants, *p73* produced NH_2_-terminally truncated forms of p73 termed the ΔNp73 variants (Δp73*α*, Δp73*β*, Δp73*γ*, Δp73*δ*, Δp73*ε*, Δp73*η*, and Δp73*ξ*) arising from the alternative promoter usage [25]. ΔNp73 lacked NH_2_-terminal transactivation domain and had an oncogenic potential [26, 27]. Indeed, higher expression levels of ΔNp73 were strongly correlated with poor prognostic outcome in neuroblastoma patients [28]. In a good agreement with these observations, ΔNp73 was frequently overexpressed in a variety of human tumor tissues as compared with their corresponding normal tissues [29]. Of note, ΔNp73 displayed a dominant-negative behavior toward TAp73 as well as wild-type p53 [25]. Furthermore, TAp73 was also inhibited by mutant forms of p53 [30]. Intriguingly, Liu et al. reported that ΔNp73*β* has an ability to induce cell cycle arrest and apoptotic cell death in association with the upregulation of p53-target genes [31]. Unlike ΔNp73*β*, ΔNp73*α* was inactive in suppressing cell growth. This complicated issue should be addressed further. 

 Under normal physiological conditions, steady-state expression of p73 is maintained at quite low level and thereby keeping this dangerous proapoptotic protein in an inactive form [7]. Initial studies showed that p73 is not induced in response to DNA damage such as actinomycin D treatment and UV exposure [1]. However, subsequent studies demonstrated that p73 is induced and activated in response to a certain subset of DNA damaging agents [32]. Accumulated evidence strongly suggests that DNA damage-mediated induction and activation of p73 is regulated by posttranslational modifications such as phosphorylation and acetylation, which is tightly linked to protein-protein interactions [5–7]. 

 In the present paper, we describe the regulatory mechanisms of proapoptotic p73 through protein-protein interactions.

## 2. Negative Autoregulatory Feedback Loop

**Figure 1:**
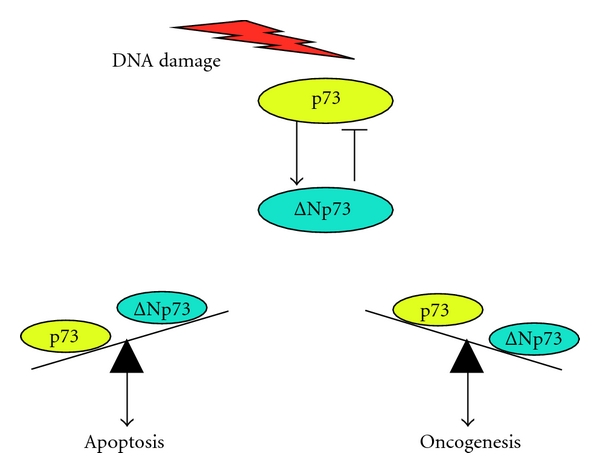
Dominant-negative effect of ΔNp73 on wild-type p73. In response to DNA damage, wild-type p73 transactivates its dominant-negative inhibitor Δ*Np73*. The intracellular balance between the expression levels of wild-type p73 and ΔNp73 is a critical determinant of cell fate.

As described in [25], ΔNp73 acts as a dominant-negative inhibitor toward wild-type p73. Since ΔNp73 had an oncogenic potential [26, 27], the balance between the intracellular expression levels of proapoptotic wild-type p73 and antiapoptotic ΔNp73 plays a critical role in the regulation of cell-fate determination. We and others found that wild-type p73 has an ability to transactivate its dominant-negative inhibitor Δ*Np73* [29, 33, 34], and thereby creating a feedback control loop which tightly regulates the proapoptotic function of wild-type p73 (Figure 1). Since ΔNp73 retains an intact central core sequence-specific DNA-binding domain as well as an oligomerization domain, it is likely that ΔNp73 inhibits wild-type p73 by direct protein-protein interaction between them through the oligomerization domain (defective hetero-oligomer formation) and/or the dominant-negative competition for DNA-binding sites with wild-type p73 [5, 35]. Thus, ΔNp73 contributes to a safety system preventing inappropriate cell death. 

 Maisse et al. demonstrated that ΔNp73 is rapidly degraded in response to DNA damage [36]. Although the precise molecular mechanisms behind the selective degradation of ΔNp73 in response to DNA damage remains elusive, this preferential degradation of ΔNp73 might relieve the inhibitory effect of ΔNp73 on wild-type p73, and results in the promotion of cell cycle arrest and/or apoptotic cell death of damaged cells. Recently, Sayan et al. found that the RING finger domain ubiquitin protein ligase termed PIR2 is a direct p73-target gene product, and PIR2 preferentially promotes the proteasomal degradation of ΔNp73 [37]. Based on their results, PIR2 expression was induced upon DNA damage in association with a significant accumulation of wild-type p73. In addition, a strong interaction between ΔNp73 and PIR2 was detectable, whereas wild-type p73 bound to PIR2 with a much lower affinity. Therefore, the preferential degradation of ΔNp73 mediated by PIR2 might be due to differential binding affinity of PIR2 to ΔNp73 and wild-type p73. 

Previously, Ohtsuka et al., described that p73 directly transactivates *cyclin G* [38]. According to their results, cyclin G bound to p73 and promoted its proteolytic degradation in a ubiquitination-independent manner. Although the precise molecular mechanisms underlying cyclin G-dependent proteolytic degradation remains unclear, there could exist a negative feedback control pathway in which cyclin G regulates the stability of p73.

## 3. Proteolytic Degradation of p73

Under normal physiological conditions, p73 is kept at an extremely low level. Since proteasome inhibitor treatment resulted in a significant accumulation of the endogenous p73 [39], p73 is regulated at least in part in a ubiquitin/proteasome-dependent manner. MDM2 which targets p53 for ubiquitin/proteasome-dependent degradation, bound to NH_2_-terminal transactivation domain of p73 but did not promote its degradation even though MDM2 inhibited p73-mediated transcriptional as well as proapoptotic activity [40–43]. It might be due to the fact that p73 lacks the degradation signal located within amino acid residues 92–112 of p53, which has been considered to confer MDM2 degradability [44]. Rossi et al. employed a phage display procedure to identify a novel p73-binding partner [45]. After the several rounds of screening, they found that HECT-type E3 ubiquitin protein ligase termed Itch is associated with p73. According to their results, their interaction was mediated by PY motif of p73 and WW domain of Itch. Itch had an ability to ubiquitinate efficiently p73 but not p53, and promoted its proteasome-dependent degradation. Itch did not interact with p53, which lacks PY motif. In contrast to p53, ΔNp73 was also the substrate of Itch. Of note, Itch was rapidly reduced in response to DNA damage through as yet unknown mechanisms, and thereby allowing p73 to increase. Asher et al. described that binding of YAP1 to PY motif of p73 prevents the interaction between Itch and p73 to stabilize p73 [46]. Consistent with these observations, Levy et al. demonstrated that YAP1 stabilizes p73 by displacing Itch binding to p73 [47]. Similarly, NEDD4-binding protein termed N4BP1 was able to interfere the complex formation of Itch with p73 [48]. N4BP1 had an undetectable effect on the stability of Itch. It appears to be important to clarify the molecular mechanisms behind DNA damage-induced downregulation of Itch. 

F-box protein termed FBXO45 contains NH_2_-terminal F-box domain and SPRY domain. SPRY domain has been shown to be involved in protein-protein interactions [49]. Recently, Peschiaroli et al. reported that FBXO45 promotes the proteasome-dependent degradation of p73 [50]. Based on their results, FBXO45 bound to p73. Their complex formation was mediated by SAM domain of p73 and SPRY domain of FBXO45. Forced expression of FBXO45 resulted in a significant reduction in expression level of p73. Treatment of proteasome inhibitor rescued FBXO45-mediated downregulation of p73, suggesting that p73 is degraded by FBXO45 in a proteasome-dependent manner. Like Itch, FBXO45 was capable to promote the ubiquitin/proteasome-dependent degradation of wild-type p73 as well as ΔNp73. Additionally, FBXO45 was down-regulated in response to DNA damage. Zhang et al. demonstrated that one of PIAS SUMO-ligase family members termed PIASy is associated with p73 and PIASy-mediated sumoylation induces proteasomal degradation of p73 [51]. PIASy significantly reduced p73-mediated transcriptional activation. Intriguingly, we have found that HECT-type E3 ubiquitin protein ligase termed NEDL2 has an ability to polyubiquitinate p73 and increase its stability [52], indicating that polyubiquitination of p73 does not always act as a degradation signal. 

 It has been shown that p73 is also regulated in a proteasome-independent manner [53]. For example, Munarriz et al. revealed that calpain I is able to cleave p73 at two distinct sites including NH_2_-terminal transactivation domain and COOH-terminal oligomerization domain [54]. Consistent with these results, forced expression of the endogenous calpain inhibitor termed calpastatin resulted in an increase in the steady-state expression level of p73.

## 4. Phosphorylation-Dependent Activation of p73

Like p53, multiple phosphorylations following DNA damage regulate the stability as well as activity of p73 [5–7]. It has been shown that DNA damage activates nonreceptor tyrosine kinase c-Abl through ATM-dependent phosphorylation of c-Abl at Ser-465 [55–59]. Initial studies demonstrated that c-Abl is associated with p73 through SH3 domain of c-Abl and PY motif of p73, and directly phosphorylates p73 at Tyr-99 in response to CDDP and ionizing radiation [60–62]. Phosphorylated form of p73 underwent nuclear redistribution and became associated with the nuclear matrix [63]. c-Abl-mediated phosphorylation of p73 at Tyr-99 increased its stability and enhanced its transcriptional as well as proapoptotic activity. It has been shown that c-Abl stimulates the catalytic activity of p38 MAP kinase [64]. Sanchez-Prieto et al. found that c-Abl-mediated activation of p38 MAP kinase leads to phosphorylation of Thr residues adjacent to Pro residues of p73, and thereby increasing the protein stability of p73 [65]. Mantovani et al. described that COOH-terminal three amino acid residues including Ser-412, Thr-442, and Thr-482 are the phosphorylation sites mediated by p38 MAPK [66]. Previous studies showed that DNA damage induces c-Abl-dependent phosphorylation of PKC*δ* and promotes the nuclear translocation of PKC*δ* [67]. Ren et al. revealed that PKC*δ* catalytic fragment (PKC*δ*CF) interacts with NH_2_-terminal transactivation domain as well as central core sequence-specific DNA-binding domain of p73, and phosphorylates p73 at Ser-289 within central core sequence-specific DNA-binding domain [68]. PKC*δ*CF-mediated phosphorylation of p73 increased the stability of p73 and enhanced its transcriptional as well as proapoptotic activity. 

 Chk1 and Chk2 have been shown to be the downstream effector kinases of ATM and ATR, which play a critical role in the regulation of DNA damage response [69, 70]. Gonzalez et al. described that Chk1 interacts with p73 and phosphorylates p73 at Ser-47 in response to DNA damage [71]. According to their results, Chk1 but not Chk2 had an ability to phosphorylate p73. Chk1-mediated phosphorylation enhanced p73-dependent transactivation capacity as well as proapoptotic function. Subsequent studies demonstrated that Chk1 and Chk2 are required for DNA damage-induced accumulation of p73 [72]. Based on their results, Chk1 and Chk2 contributed to the transcriptional activation of *p73* gene, which might be due to the stabilization of E2F1 in response to DNA damage. As described previously [73–75], E2F1 acts as a transcription factor for *p73*. 

 It has been shown that c-Abl activates the JNK signaling pathway in response to DNA damage, and JNK is closely involved in the apoptotic response to a variety of genotoxic stresses [56, 76, 77]. Recently, Jones et al. reported that JNK has an ability to stabilize p73 in a phosphorylation-dependent manner [78]. JNK was associated with p73 and phosphorylated its multiple Ser and Thr residues including Ser-8, Ser-97, Ser-110, Ser-333, Ser-412, Thr-442, and Thr-482. JNK-mediated phosphorylation enhanced the transcriptional as well as proapoptotic activity of p73. 

 Previously, we described that COOH-terminal Pro-rich domain (amino acid residues 380–513) contains transactivation ability as examined by GAL4 system [79]. Recently, Nyman et al. precisely identified the COOH-terminal transactivation domain of p73 which exists within amino acid residues 381 to 399 [80]. Intriguingly, this second COOH-terminal transactivation domain preferentially regulated the transcription of p53-target genes implicated in the induction of cell cycle arrest rather than apoptotic cell death. PKC phosphorylated p73 at Ser-388, and markedly enhanced the transcriptional activity of p73. Mutation at Ser-388 resulted in a remarkable reduction of the p73-mediated transcriptional activation. Taken together, their results indicate that PKC-mediated phosophorylation of p73 at Ser-388 contributes to the selective induction of cell cycle arrest. 

**Figure 2:**
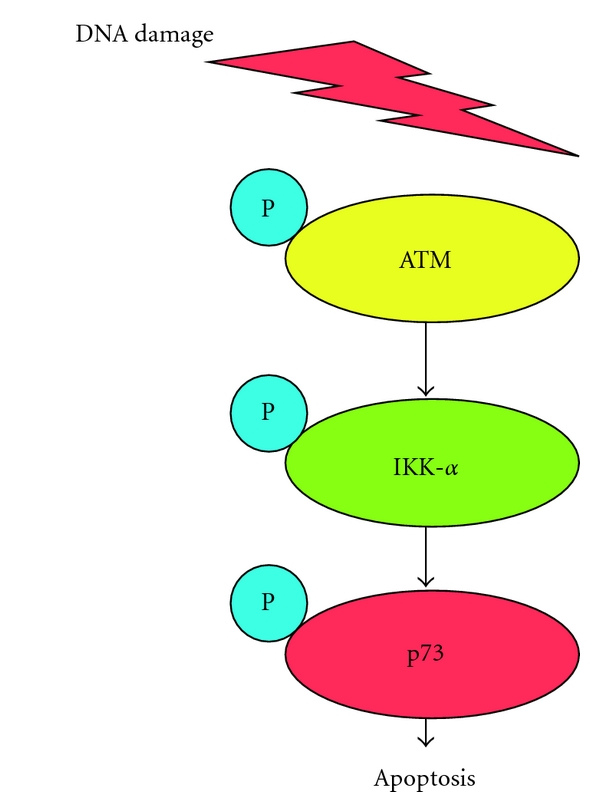
p73-dependent apoptotic cell death in response to DNA damage. Upon DNA damage, phospho-ATM phosphorylates IKK-*α* and promotes nuclear accumulation of IKK-*α*. IKK-*α* then enhances transcriptional as well as proapoptotic function of p73 in a p53-independent manner.

 It has been well known that IKK (I*κ*B kinase) complex acts as an upstream regulator of prosurvival NF-*κ*B signaling pathway [81]. IKK complex is composed of three functional subunits including IKK-*α*, IKK-*β*, and IKK-*γ*. We have found that nuclear IKK-*α* is induced to accumulate in response to DNA damage and associated with the central core sequence-specific DNA-binding domain of p73 in nuclear matrix [82]. DNA damage-mediated nuclear accumulation of IKK-*α* was dependent on phospho-ATM [83]. IKK-*α* had an ability to stabilize p73 by inhibiting its polyubiquitination, and enhanced p73-mediated transcriptional activation as well as proapoptotic activity. In addition, IKK-*α* phosphorylated the NH_2_-terminal portion of p73 and a kinase-deficient mutant form of IKK-*α* had undetectable effect on p73. Thus, IKK-*α*-mediated activation of p73 was regulated in a phosphorylation-dependent manner (Figure 2).

## 5. Phosphorylation-Dependent Inhibition of p73

Phosphorylation does not always act as an activation signal. Irwin et al. described that expression of p73 is regulated in a cell cycle-dependent manner [74]. Under their experimental conditions, p73 started to increase at the end of G1 phase, which was mediated by E2F1. Gaiddon et al. found that p73 interacts with cyclin/CDK complex through its cyclin recognition motif (CRM) [84]. p73 contains two CRM sequences located at NH_2_-terminal portion (149-KKL-151) and COOH-terminal region (515-RAL-517). Cyclin/CDK complex phosphorylated p73 at Thr-86 and p73 was most highly phosphorylated at Thr-86 in G2 as well as M phase. From the functional point of view, cyclin/CDK-mediated phosphorylation at Thr-86 reduced the transcriptional activity of p73. However, the biological significance of phospho-p73 in the regulation of cell cycle progression remains unclear. 

 During the extensive search for p73-binding partners by using a yeast-based two-hybrid screening procedure, we have identified PKA catalytic subunit *β* (PKA-C*β*) as a novel p73-binding protein [85]. p73 bound to PKA-C*β* in mammalian cells through its NH_2_-terminal portion (amino acid residues 63–130) and COOH-terminal region (amino acid residues 469–636). *In vitro* kinase reactions revealed that PKA-C*β* is able to phosphorylate NH_2_-terminal region of p73. Within this region, there exists a putative PKA recognition site (78-RAAS-82). Of note, PKA-C*β* inhibited p73-mediated transcriptional activation as well as proapoptotic activity following DNA damage in a phosphorylation-dependent manner. 

 Hck is one of the nonreceptor tyrosine kinase Src family [86–88]. Considering that Hck interacts with c-Abl and their interaction modulates each other's activity [89], Paliwal et al. investigated the possible role of Hck in the regulation of p73 [90]. According to their results, Hck was associated with p73 through its SH3 domain, and phosphorylated p73 at Tyr-28. Forced expression of Hck led to the stabilization of cytoplasmic p73 but not of nuclear p73 in a phosphorylation-dependent manner. Hck inhibited p73-mediated transcriptional as well as proapoptotic activity, which required the intact SH3 domain of Hck, suggesting that physical interaction between Hck and p73 plays an important role in the inhibition of p73. 

 Plk (Polo-like kinase) family is composed of four members including Plk1, Plk2, Plk3, and Plk4. Plk1 has been considered to be a positive cell cycle regulator [91–93], and also shown to be a negative regulator of p53 [94]. Recently, we have found for the first time that Plk1 attenuates transcriptional as well as proapoptotic function of p73 [53]. Based on our results, Plk1 was associated with p73 through NH_2_-terminal domain of p73 and catalytic domain of Plk1. Kinase-deficient Plk1 had a marginal effect on p73. Indeed,* in vitro* kinase reactions demonstrated that Plk1 phosphorylates p73 at Thr-27. Komatsu et al. revealed that Plk1 also suppresses another p53 family member, p63, through physical interaction and phosphorylation [95]. Plk1 phosphorylated p63 at Ser-52. 

 Plk3 has been shown to be closely involved in the regulation of DNA damage response through the direct interaction with p53 accompanied with an induction of its phosphorylation at Ser-20 [96, 97]. In contrast to Plk3/p53 interaction, we have demonstrated that Plk3 is associated with NH_2_-terminal region of p73 and inhibits p73-mediated transacriptional activation as well as proapoptotic activity [98]. In addition, Plk3 decreased the stability of p73. Since kinase-deficient Plk3 had an undetectable effect on p73, Plk3-mediated inhibition was regulated in a phosphorylation-dependent manner. In fact, Plk3 was able to phosphorylate certain amino acid residue(s) between 63 and 113 of p73. Thus, it is likely that Plk3 attenuates the inappropriate p73-mediated apoptotic cell death in response to DNA damage.

## 6. Acetylation-Dependent Regulation of p73

Like p53, p73 is also regulated by p300-mediated acetylation. Zeng et al. showed that histone acetyltransferase p300 binds to p73 through its NH_2_-terminal CH1 domain and NH_2_-terminal transactivation domain of p73 [99]. p300 stimulated p73-mediated transcriptional activation as well as proapoptotic function. Subsequent studies demonstrated that p73 is acetylated by p300 at Lys-321, Lys-327 and Lys-331 in response to DNA damage [100]. Furthermore, c-Abl was required for DNA damage-mediated acetylation of p73 and acetylated forms of p73 preferentially transactivated p53-target genes implicated in the induction of apoptotic cell death such as *p53AIP1*. Mantovani et al. found that prolyl isomerase Pin1 promotes the conformational change of p73, and enhances proapoptotic activity of p73 [66]. According to their results, c-Abl enhanced the phosphorylation-dependent interaction between Pin1 and p73, and thereby inducing the acetylation of p73 mediated by p300. Indeed, Pin1 greatly enhanced p73 acetylation by p300. In addition, forced expression of p300 resulted in a remarkable stabilization of p73. Strano et al. revealed that YAP1 promotes p73/p300 complex formation and potentiates p300-mediated acetylation of p73 [101]. Therefore, it is likely that p300-mediated acetylation of p73 reduces the ubiquitination levels of p73 by competition between acetylation and ubiquitination. In support with these observations, SIRT1 with an intrinsic deacetylase activity abrogated p73-mediated transacriptional activation and apoptotic cell death [102]. On the other hand, Zeng et al. demonstrated that p300 acts as a coactivator of p73 without inducing its acetylation [103]. Further studies should be required to address this issue.

## 7. Subcellular Localization of p73

Subcellular localization is one of the critical determinants for the activity of p73. Kim et al. identified HIPK2 as a novel p73-binding partner by using yeast-based two-hybrid screening [104]. HIPK2 has been shown to be Ser/Thr kinase which phosphorylates p53 at Ser-46 and enhance proapoptotic activity of p53 [105]. HIPK2 bound to p73 through its COOH-terminal region containing PEST sequence and the oligomerization domain of p73. Reporter assays revealed that HIPK2 enhances the transcriptional activity of p73. Intriguingly, HIPK2 colocalized with p73 in the nuclear body, suggesting that nuclear structures such as nuclear body might provide an important subnuclear locale for p73 function. 

 Previously, Kim et al. employed the yeast-based two-hybrid procedure to identify a novel p73-binding protein [106]. After the extensive screening, they identified amphiphysin IIb-1. Amphiphysin IIb-1 which contains NH_2_-terminal BAR domain and COOH-terminal SH3 domain, was one of the splicing variants of cytoplasmic amphiphysin IIb [107]. According to their results, amphiphysin IIb-1 interacted with p73 through its COOH-terminal SH3 domain and COOH-terminal region of p73 (amino acid residues 321–376). This interaction significantly inhibited p73-mediated transacriptional activation and apoptotic cell death. Subsequent studies demonstrated that amphiphysin IIb-1 promotes the cytoplasmic relocalization of p73 by masking the nuclear localization signal (NLS) of p73 (amino acid residues 338–348).

*WWOX* gene is located at 16q23.2-24.1, a genomic region with a high incidence of loss of heterozygosity (LOH) and homozygous deletions [108]. Ectopic expression of WWOX in breast cancer cells inhibited tumor growth *in vivo* [109], indicating that WWOX might be a candidate tumor-suppressor. Since WWOX contains two WW domains, Fabbri et al. performed the affinity assays to search for cellular proteins which could bind to WW domain of WWOX [110]. Finally, they found that PY motif of p73 interacts with WW domain of WWOX. In contrast to p73, WWOX failed to bind to p53 which lacks PY motif. Based on their results, WWOX caused the redistribution of p73 from nuclear compartment to the cytoplasm and repressed the transactivation function of p73. Of note, cytoplasmic p73 enhanced proapoptotic activity of WWOX in a transcription-independent manner. However, the precise molecular mechanisms behind the cytoplasmic p73-mediated activation of WWOX remains obscure.

## 8. Positive Regulation of p73 through Protein-Protein Interaction

Protein-protein interaction is one of the central events in a variety of cellular biological response. In this connection, p73 is regulated by protein-protein interactions.

By using the extensive pull-down assays, Strano et al. identified YAP1 as one of p73-binding proteins [111]. YAP1 was originally identified as an adaptor protein binding to SH3 domain of the Yes proto-oncogene product belonging to the Src family of protein tyrosine kinases. YAP1 bound to PY motif of p73 through its WW domain. YAP1 was not associated with p53. Introduction of the mutation into PY motif of p73 abrogated the interaction between p73 and YAP1, suggesting that structural integrity of PY motif is necessary for the interaction with YAP1. Forced expression of YAP1 enhanced transactivation ability of p73, indicating that YAP1 acts as a coactivator for p73. 

**Figure 3:**
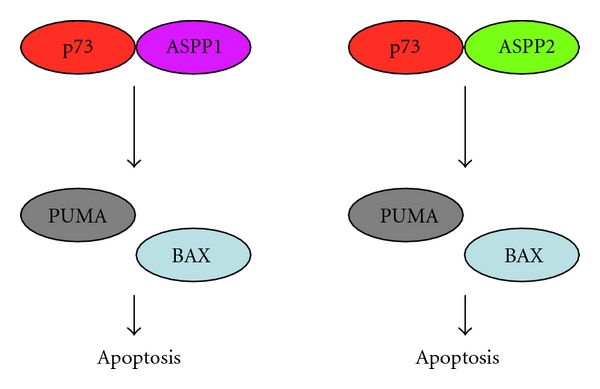
p73/ASPP complex preferentially induces the expression of proapoptotic BAX and PUMA. p73 forms a complex with ASPP1 or with ASPP2, and these transcriptional complexes selectively transactivate proapoptotic p73-target genes such as *BAX* and *PUMA*.

 As described previously [112], ASPP1 and ASPP2 belonging to ASPP family, bound to the central core sequence-specific DNA-binding domain of p53, and specifically stimulated the transcription of p53-target genes implicated in the induction of apoptotic cell death. Bergamaschi et al. examined the possible effect of ASPP1 and ASPP2 on p73 [113]. According to their results, ASPP1 and ASPP2 were able to interact with p73, and selectively enhanced p73-mediated transactivation of proapoptotic *BAX*, *PIG3,* and *PUMA* genes but not of *p*21^WAF1^ and *MDM2* genes. Consistent with these observations, ASPP1 and ASPP2 enhanced proapoptotic function of p73 in response to DNA damage. It is likely that ASPP1 and ASPP2 are closely involved in the regulation of promoter selectivity of p73 (Figure 3). 

Jeong et al. identified p19^ras^ as a novel p73-binding protein by using the yeast-based two-hybrid screening [114]. p19^ras^ is an alternative splicing variant of c-H-ras, and lacks transforming potential [115]. Systematic deletion analysis revealed that p19^ras^ interacts with p73 DNA-binding domain-containing region (amino acid residues 54–310). Of note, p19^ras^ remarkably disrupted the complex formation of p73 with its negative regulator MDM2. As expected, p19^ras^ blocked MDM2-mediated transcriptional repression of p73 and led to the activation of p73. 

 Similarly, we have employed the yeast-based two-hybrid screening to identify p73-binding proteins. After several rounds of extensive screening, we have obtained MM1 and RanBPM [116, 117]. MM1 has been shown to interact with c-Myc and suppress its transactivation function [118]. MM1 bound to COOH-terminal region of p73 (amino acid residues 551–636) in cell nucleus. In contrast to p73, MM1 failed to interact with p53. Forced expression of MM1 resulted in an enhancement of p73-mediated transcriptional as well as growth-suppressing activity. As expected, MM1 had an undetectable effect on p53. RanBPM was initially identified as a cellular protein which interacts with Ran nuclear-cytoplasmic transport protein [119, 120], and contains the putative SPRY domain which might be involved in protein-protein interactions. The region containing the canonical SPRY domain of RanBPM was associated with COOH-terminal region of p73 (amino acid residues 551–636). Like MM1, RanBPM did not bind to p53. RanBPM had an ability to stabilize p73 by inhibiting its ubiquitination. Finally, RanBPM enhanced transcriptional and proapoptotic activities of p73.

## 9. Negative Regulation of p73 through Protein-Protein Interactions

As described previously [121, 122], *WT1* gene mapped at human chromosome 11p13 has been considered to be a candidate tumor-suppressor gene for Wilm's tumor. Subsequent studies demonstrated that WT1 contains zinc finger domain and acts as a sequence-specific transcription factor [123]. Scharnhorst et al. reported that WT1 interacts with p73 through its zinc finger domain [124]. WT1 had a negligible effect on the stability of p73 but strongly inhibited p73-mediated transcriptional activation. 

 Tax which is encoded by the human T-cell leukemia virus type I (HTLV-I), has been shown to be HTLV-I-associated malignant transformation [125, 126]. Indeed, Tax induced tumorigenesis and leukemogenesis in mice [127]. Although Tax had an ability to stabilize p73, p73-mediated transcriptional activation was significantly inhibited by Tax [128]. Since CBP with an intrinsic histone acetyl transferase activity acts as transcriptional coactivator for Tax and p73 [99, 129], Tax-mediated repression of p73 might be due to the competition between Tax and p73 for CBP. Thus, it is likely that inactivation of p73 by Tax contributes to HTLV-I-dependent leukemogenesis. 

 Hepatitis C virus (HCV) core protein has been shown to modulate p53 [130, 131]. Alisi et al. found that HCV core protein directly interacts with COOH-terminal portion of p73 (amino acid residues 321–353) [132]. Luciferase reporter assays demonstrated that HCV core protein enhances the p73-dependent luciferase activity driven by *MDM2* promoter, whereas p73-dependent transactivation of *BAX* is significantly inhibited by HCV core protein. Consistent with these observations, HCV core protein almost completely blocked the ability of p73 to reduce cell viability. 

 It has been shown that adenovirus E1B, SV40 large T antigen and human papilloma virus E6 which inhibit p53, do not interact with p73 and do not affect p73 activity [133, 134]. Similarly, adenovirus E4orf6 protein with oncogenic potential bound to COOH-terminal region of p53 and inhibited transcriptional activity of p53 [135]. Higashino et al. reported that E4orf6 is also associated with COOH-terminal region of p73 and blocks p73-mediated transcriptional as well as proapoptotic activity [136]. Thus, it is possible that E4orf6-mediated inhibition of p53 and p73 is responsible for the enhanced tumorigenic potential of adenovirus. Additionally, these findings suggest that only a subset of viral oncoproteins interact with p73. 

 Nagatani et al. found that the CCAAT-binding transcription factor CTF2 is overexpressed in CDDP-resistant cells, suggesting that CTF2 is involved in the acquisition of drug-resistant phenotype of tumor cells [137]. Subsequent studies revealed that CTF2 interacts with central core sequence-specific DNA-binding domain of p73 (amino acid residues 228–312) [138]. According to their results, CTF2 strongly inhibited the sequence-specific DNA-binding activity of p73. Therefore, it is likely that CTF2-dependent inhibition of p73 contribute to the acquisition of drug-resistant phenotype of tumor cells. 

 Bcl-2 associated athanogene BAG-1 protected cells from cellular stress-mediated apoptotic cell death [139–141]. In accordance with these results, BAG-1 expression correlated with important clinical parameters of certain tumors [142]. Wang et al. reported that BAG-1 is associated with p73 through its intact BAG domain, and strongly attenuates transcriptional activity of p73 [143]. Knocking down of the endogenous BAG-1 resulted in a reactivation of p73. Additionally, BAG-1 reduced the stability of p73. Collectively, BAG-1-mediated inhibition of p73 might be one of the molecular mechanisms by which BAG-1 interfered with stress-induced apoptotic cell death. 

 TIP60 is a member of MYST family of histone acetyltransferase which is conserved from yeast to human [144]. It has been shown that TIP60 acts as a coactivator for p53 by inhibiting MDM2-mediated proteolytic degradation of p53 [145]. TIP60-mediated acetylation of p53 at Lys-120 had a critical role in the regulation of p53-dependent apoptotic response [146, 147]. Recently, Kim et al. described that, in contrast to p53, TIP60 strongly inhibits transcriptional as well as proapoptotic function of p73 [148]. TIP60 formed a ternary complex with p73, which was bridged by MDM2. It is likely that TIP60 recruits MDM2 onto p73 thereby inhibiting the activity of p73. 

 It is worth noting that human tumor-derived p53 mutants strongly inhibit p73 [30]. As described previously [149], forced expression of p53 mutant caused increased chemoresistance to anticancer drugs, suggesting that mutant p53 inactivates p73-induced apoptosis through the interaction with p73. Strano et al. found that mutant p53 interacts with p73 through its central core sequence-specific DNA-binding domain and p73 region containing central core sequence-specific DNA-binding domain and oligomerization domain [150]. Consistent with the previous observations [30], This interaction markedly suppressed transcriptional activity of p73. Since mutant p53 bound to the oligomerization domain of p73, homotetramer formation of p73 might be blocked by mutant p53. Alternatively, binding of mutant p53 to central core sequence-specific DNA-binding domain of p73 might attenuate its binding to p53/p73-responsive element. Sang et al. described that forced expression of p73 induces apoptotic cell death in human breast cancer-derived and chemo-resistant MDA-MB-436 cells bearing *p53* mutation [151], indicating that the balance between the intracellular expression levels of mutant p53 and p73 is a critical determinant of chemosensitivity.

## 10. Future Perspective

**Figure 4:**
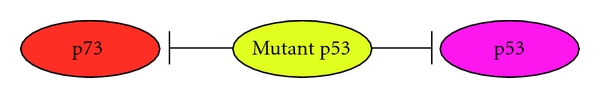
Dominant-negative behavior of mutant p53 toward wild-type p73 and p53. Mutant form of p53 binds to wild-type p73 as well as p53 and strongly inhibits their tumor suppressive function.

As described in the present paper, a variety of cellular and viral proteins are involved in the positive and negative regulations of proapoptotic p73 through protein-protein interactions. From the clinical point of view, over 50% human tumors carry *p53* mutations. In some cases, tumors bearing *p53* mutations displayed chemoresistant phenotype. On the other hand, *p73* was infrequently mutated in human tumors. Therefore, it is quite important to clarify the precise molecular mechanisms underlying p73-dependent apoptotic pathway in response to DNA damage. Furthermore, mutant p53 acts as a dominant-negative inhibitor toward p73 as well as p53 (Figure 4). In this connection, development of a novel strategy to overcome and/or eliminate the dominant-negative effect of mutant p53 on p73 and p53 is required for the effective treatment of tumors carrying *p53 *mutations.
